# Mechanism and Prevention of Ototoxicity Induced by Aminoglycosides

**DOI:** 10.3389/fncel.2021.692762

**Published:** 2021-06-15

**Authors:** Xiaolong Fu, Peifeng Wan, Peipei Li, Jinpeng Wang, Siwei Guo, Yuan Zhang, Yachun An, Chao Ye, Ziyi Liu, Jiangang Gao, Jianming Yang, Jiangang Fan, Renjie Chai

**Affiliations:** ^1^State Key Laboratory of Bioelectronics, Jiangsu Province High-Tech Key Laboratory for Bio-Medical Research, School of Life Sciences and Technology, Southeast University, Nanjing, China; ^2^School of Life Science, Shandong University, Qingdao, China; ^3^Department of Otology, The First Affiliated Hospital of Zhengzhou University, Zhengzhou, China; ^4^The Key Laboratory of Animal Resistant Biology of Shandong, College of Life Science, Shandong Normal University, Jinan, China; ^5^Second Hospital of Anhui Medical University, Hefei, China; ^6^Department of Otolaryngology Head and Neck Surgery, Sichuan Academy of Medical Science, Sichuan Provincial People's Hospital, Chengdu, China; ^7^Co-Innovation Center of Neuroregeneration, Nantong University, Nantong, China; ^8^Institute of Stem Cell and Regeneration, Chinese Academy of Sciences, Beijing, China; ^9^Beijing Key Laboratory of Neural Regeneration and Repair, Capital Medical University, Beijing, China

**Keywords:** ototoxicity, aminoglycosides, hearing loss, mechanism, prevention

## Abstract

Aminoglycosides, a class of clinically important drugs, are widely used worldwide against gram-negative bacterial infections. However, there is growing evidence that aminoglycosides can cause hearing loss or balance problems. In this article, we mainly introduce the main mechanism of ototoxicity induced by aminoglycosides. Genetic analysis showed that the susceptibility of aminoglycosides was attributable to mutations in mtDNA, especially A1555G and C1494T mutations in 12S rRNA. In addition, the overexpression of NMDA receptors and the formation of free radicals also play an important role. Understanding the mechanism of ototoxicity induced by aminoglycosides is helpful to develop new therapeutic methods to protect hearing. In this article, the prevention methods of ototoxicity induced by aminoglycosides were introduced from the upstream and downstream aspects.

## Introduction

Hearing loss is the most common sensory disorder worldwide. The World Health Organization estimates that around 466 million people worldwide suffer from disabling hearing loss and that by 2050 more than 900 million people will have this condition. Disabled hearing loss is defined as a loss of more than 40 dB in the better-hearing ear of an adult and more than 30 dB in the better-hearing ear of a child. Hearing loss can affect an individual's quality of life, making it difficult to understand speech in life. And untreated hearing loss costs the world an additional $750 billion a year.

Hearing loss has both congenital and acquired caused. Congenital causes are caused by hereditary or non-hereditary factors. Acquired deafness can be caused by noise, ear infections, age, and medication. It is worth noting that ototoxic drugs are an important factor in the induction of hearing loss. Ototoxic drugs mainly affect hair cells, causing permanent damage to hearing. Because hair cells in mammals are terminally differentiated and do not have the ability to regenerate spontaneously if they die, ototoxic drugs can have serious effects on hearing (Forge et al., 1993). Ototoxic drugs mainly include aminoglycoside antibiotics, non-aminoglycoside antibiotics, antitumor drugs, and salicylate, etc. Aminoglycoside antibiotics are one of the early antibiotics used in the treatment of bacterial infections, and are widely used in the world, especially in developing countries. In this article, we introduce the mechanism of ototoxicity induced by aminoglycosides and propose some prevention strategies.

## Aminoglycosides

Some therapeutic drugs used to treat life-threatening conditions, such as aminoglycosides and antitumor drugs, can cause hearing loss and/or balance problems (Guo et al., 2019). Antibiotics are widely used in clinical practice, and more than 100 kinds of ototoxic drugs have been found. Aminoglycosides have been shown to be highly effective against gram-negative infections (Gao et al., 2017). Aminoglycosides are glycosides formed from amino sugars and aminocyclitol linked by an oxygen bridge. Aminoglycosides are bactericide that inhibit protein synthesis and have a wide antibacterial spectrum and strong antibacterial activity. They are commonly used in clinical practice. Although aminoglycosides are highly effective and relatively inexpensive, they are known to have ototoxicity and vestibular toxicity. Aminoglycosides can accumulate in the inner ear and are difficult to metabolize, leading to permanent hearing loss (Fischel-Ghodsian, 2005). The most common aminoglycoside drugs are streptomycin, gentamicin, neomycin, it's long-term use will lead to neurological tinnitus, neurological deafness, and even memory loss, hearing loss, dizziness and other conditions. The use of antibiotics (especially aminoglycoside antibiotics) has become the main cause of hearing loss in children in China, and both topical and systemic drugs can lead to hearing loss.

## Mechanism of Aminoglycoside Ototoxicity

Clinically, aminoglycoside drugs mainly enter the inner ear through systemic and topical pathways. In the systemic pathway, the drug passes through the blood-labyrinth barrier (BLB) and enters the inner ear through the stria vascularis. In topical administration, the drug can bypass the BLB into the middle ear and then through the round window into the inner ear. The drug is absorbed either by endocytosis on the apical surface (Hashino and Shero, 1995) or by transduction channels (Marcotti et al., 2005). Early genetic analysis showed that the susceptibility of aminoglycosides was related to mitochondrial DNA mutations, which inhibited the synthesis of mitochondrial proteins. However, evidence is accumulating to suggest that the overactivation of N-methyl-D-aspartate (NMDA) receptors and the production of free radicals are also important factors in the ototoxicity of aminoglycosides (Strupp and Arbusow, 2001). The mechanism of aminoglycosides-induced ototoxicity is very subtle. How does aminoglycoside cause hearing loss has been a hot research topic.

### Associations Between Susceptibility to Aminoglycosides and Mitochondrial DNA Mutations

Mitochondria are energy providers and mediators of cell apoptosis, which play an important role in cell survival. Aminoglycoside susceptibility has been reported to be related to genetic background in many individuals. The mammalian mitochondria genome is maternally inherited. Interestingly, lineage analysis suggests that aminoglycosides-induced hearing loss is also maternally inherited (Prezant et al., 1993; Rydzanicz et al., 2010). It is suggested that aminoglycoside sensitivity is related to mitochondria. Mitochondria have their own genome, called mtDNA, which encodes 22 tRNAs, 13 mRNAs, and 2 rRNAs, which are important for the composition of the OXPHOS respiratory chain complex (Kokotas et al., 2007). Studies have found that when mitochondrial DNA (especially 12S rRNA) is mutated, it increases the binding to aminoglycosides, inhibits the synthesis of mitochondrial proteins and increases the formation of free radicals, which in turn affects hearing (Qian and Guan, 2009). This suggests that mitochondrial DNA mutations are associated with susceptibility to aminoglycosides.

#### Mitochondria

Mitochondria are closely related to cell survival. There areresponsible for the production of cellular adenosine triphosphate (ATP), which provides organisms with the energy necessary for survival, and for the regulation of important function, including apoptosis and the production of free radicals (Van Remmen and Jones, 2009). They have their own DNA called mtDNA, which codes for mitochondria proteins. MtDNA accounts for about 0.5% of the total DNA in a nucleated somatic cell (Kokotas et al., 2007). The mtDNA molecule is a double stranded, circular structure with a total length of 16,568 bp, encoding 22 tRNAs, 13mRNAs, 2rRNAs (Kokotas et al., 2007). The 13 mitochondrial proteins are involved in the formation of the OXPHOS respiratory chain complex. Once the translation defect or dysfunction of several components of OXPHOS occurs, it may affect the generation of energy, thus affecting the survival state of cells and causing a variety of diseases (Kawamata and Manfredi, 2017). Another important function of the mitochondria is to produce free radicals. Free radicals can be produced in a variety of ways, of which mitochondria are the main source. In normal metabolic processes, 1–4% of oxygen is incompletely reduced, resulting in the production of ROS (Zorov et al., 2014). In addition, the monoamine oxidase in the outer membrane of mitochondria and the α-ketoglutarate dehydrogenase complex in the matrix can also produce ROS (Starkov, 2013).

In eukaryotes, ribosomes have also been detected in mitochondria in addition to the cytoplasm. Interestingly, mammalian mitochondria ribosomes are more similar to prokaryotes 70S ribosomes (Collatz et al., 1976). According to the “endosymbiotic theory,” mitochondria are derived from bacteria, that is, after bacteria are swallowed by eukaryotes, in the long-term symbiosis process, they form mitochondria through evolution (Martin et al., 2015). According to this theory, the ancestral mitochondria (a kind of gram-negative bacteria that can carry out the tricarboxylic acid cycle and electron transfer) were swallowed by the primitive eukaryotes and formed a symbiotic relationship with the host. The mammalian mitochondrial ribosome is a 55S protein complex composed of two subunits, 28S and 39S, which has the function of translating mitochondrial mRNA encoded by mtDNA (Collatz et al., 1976). What's more, the mtDNA encodes 12S ribosomal RNA. When mitochondrial ribosomes are damaged, it will lead to impaired synthesis of mitochondrial protein, affect the generation of cellular energy, and cause damage to the survival state of cells.

#### Aminoglycosides Destroy Bacteria

Aminoglycosides are widely used to fight gram-negative bacterial infections. Early studies have found that aminoglycosides act on the 30S subunits of bacterial ribosomes, causing bacterial code reading errors and eventually causing bacterial death. The prokaryotic ribosomes are composed of 30S and 50S subunits, while the eukaryotic ribosomes are composed of 40S and 60S subunits (Wimberly et al., 2000). Because of the structural difference between the 70S and 80S ribosomes of bacteria, aminoglycosides kill bacteria without destroying the infected cells (Gutell et al., 1994). In recent years, more in-depth studies have found that aminoglycosides directly bind to the A site of the 16S rRNA. Interestingly, in mammalian mitochondria, the A site on the aminoglycoside binding 16S rRNA is replaced by the G site (Huth et al., 2015). This may account for the difference between the effects of aminoglycosides on mammals and bacteria.

#### Associations Between Mitochondrial DNA Mutations and Aminoglycosides

Mitochondria have their own genomes, encoding mitochondrial proteins. Previous studies have found that mtDNA has a higher mutation rate than nuclear DNA and accumulates in cells with age, with a mutation at least 10-fold than in nuclear DNA (Wallace et al., 1987). Moreover, mitochondrial DNA has a poorer repair mechanism than nuclear DNA. Mitochondria play a very important role in the survival of cells. When mitochondrial function is abnormal, it will cause serious physiological dysfunction. MtDNA plays an important role in maintaining mitochondrial function. MtDNA mutations might lead to both multisystem disorders, such as Leber hereditary optic neuropathy (LHON); myoclonus epilepsy associated with ragged-red fibers (MERRF); or non-syndromic deafness (Kokotas et al., 2007). Mutations in some mtDNA that encode rRNA or tRNA have been found to cause non-syndromic hearing loss. It is mainly due to the mutation of mitochondrial ribosomal small subunits, especially A1555G and C1494T are primary genetic characteristics of the mutation, and susceptible to aminoglycoside-induced hearing loss (Zhao et al., 2004; Young et al., 2005; Bravo et al., 2006).

The A1555G mutation is the most common type of mitochondrial 12S rRNA mutation in aminoglycoside-induced hearing loss (Bravo et al., 2006). The A1555G alone is not sufficient to induce a clinical phenotype, and there may be other factors that co-regulate the effects of A1555G mutations on hearing. The A1555G mutation-associated deafness penetrance may be regulated by aminoglycosides, nuclear modifiers genes, or other mtDNA mutations (Guan et al., 1996, 2000). It was reported that the homoplasmic A1555G mutation at the A site of highly conserved 12S rRNA have been associated with aminoglycoside-induced non-syndromic hearing loss in many families worldwide (Li et al., 2004; Young et al., 2005; Yuan et al., 2005). When the 1555A is mutated to G, the secondary structure of 12S rRNA more closely resembles the corresponding region of 16S rRNA in bacteria. Thus, the A1555G mutation may alter the secondary structure of 12S rRNA, leading to increased susceptibility of aminoglycosides (Kokotas et al., 2007).

The second mutation, the C1494T mutation of the mitochondrial 12S rRNA gene. Sequence analysis of mitochondria DNA from a large Chinese family of aminoglycoside-induced hearing loss revealed the C1494T mutation in the 12S rRNA gene (Zhao et al., 2004). The site of 1,494 is the corresponding site of 1,555 located at 12S rRNA highly conserved A-site (Zhao et al., 2004).

Complete mitochondrial genome sequence analysis of individuals revealed that aminoglycoside drug sensitivity was associated with several other mitochondrial DNA mutations. Mitochondrial genome mutation analysis revealed the presence of homoplasmic 12S rRNA A827G mutation in the patient's mitochondria, which is associated with hearing loss (Chaig et al., 2008). The A827G mutation may lead to tertiary or quaternary structural changes in the 12S rRNA that affect mitochondrial function, thus playing an important role in aminoglycosides-induced hearing loss (Chaig et al., 2008). The study found that when individuals with the T1095C mutation were exposed to aminoglycoside antibiotics, the number of apoptotic cells in the mutant individuals were ten-fold higher than in the control group. These results indicate the pathogenicity of the T1095C mutation, which increases the susceptibility of aminoglycoside (Muyderman et al., 2012). Furthermore, mutations such as A745G, C792T, A801G, A856G, A1027G, C1192T, C1310T and A1331G may be related to the aminoglycoside ototoxicity (Lu et al., 2010). ADDIN EN.CITE (Hong et al., 2006).

### NMDA Is Involved in Ototoxicity Induced by Aminoglycosides

The N-methyl-D-aspartate (NMDA) is a glutamate receptor, which exists at the synaptic site between cochlear hair cells and the radical dendrites of spiral ganglion afferents. Aminoglycosides may mimic the effects of polyamines on NMDA receptors (Puel, 1995). The association between aminoglycosides and polyamines may explain the glutamate-like excitotoxicity induced by aminoglycosides. The overstimulation of NMDA receptors (NMDARs) increases the formation of nitric oxide (NO), resulting in oxidative stress on hair cells. In addition, some studies have shown that gentamicin treatment can increase the expression of nNOS and iNOS to induce hair cell injury (Hong et al., 2006; Jia et al., 2018). More importantly, high doses of aminoglycosides may increase the entry of calcium ions through NMDA-related channels (Bienkowski et al., 2000). Increased influx of calcium ions is the basis for formation of excitotoxicity. Excitotoxicity can be produced by a two-step mechanism: The first is acute swelling, which accompanies the destruction of postsynaptic structure, followed by a cascade of calcium ions that leads to the death of neuron (Pavlidis et al., 2014). In the experiment, after intramuscular injection of amikacin, the amplitude of DPOAE was significantly different from that of the control group (Pavlidis et al., 2014). More importantly, NMDA receptors antagonists can prevent ototoxicity caused by aminoglycosides and treat hearing loss (Basile et al., 1996; Pavlidis et al., 2014). It is interesting to note the influx of calcium through NMDARs may induce immediate transcription of early genes through mitogen-activated protein kinases (MAPK)-dependent mechanisms (Xia et al., 1996). Substrates of ERK and JNK subfamilies of MAPK, c-Fos and c-Jun, form AP-1 transcription factor complexes (Xia et al., 1996). It can be seen from the above studies that NMDA plays an important role in the ototoxicity induce by aminoglycosides, which is worth further exploration.

### ROS Is Involved in Ototoxicity Induced by Aminoglycosides

It is generally accepted that ROS is involved in ototoxicity induced by aminoglycosides, and antioxidants can mitigate the effects of ototoxicity (Xie et al., 2011). Aminoglycosides have also been reported to produce free radicals in the inner ear that subsequently cause damage to sensory cells and neurons, leading to hearing loss (Rybak and Kelly, 2003). ROS is a normal product in the metabolic of organisms. It plays a role in regulating messengers in various processes such as proliferation, survival, gene expression and apoptosis, and is also a signal molecule for homeostasis adaptation under stress conditions (Finkel, 2012). Under normal circumstances, ROS are easily cleared by antioxidants in the body, such as catalase, superoxide dismutase (SOD) and glutathione, to prevent ROS from escaping and entering cells, so as to maintain homeostasis in the inner ear (Kopke et al., 1999; Bared et al., 2010). However, aging, drugs, the environment and other factors can change this balance. ROS can be generated by, NADPH oxidase and mitochondrial, peroxisomal, or microsomal pathways (Bottger and Schacht, 2013). In addition, aminoglycosides can combine with transition metals such as iron and copper to form free radicals (Schacht, 1999; Dehne et al., 2002). It has been reported that Fe II-aminoglycoside complexes can combine with phosphatidylinositol and induce the release of arachidonic acid. At the same time, arachidonic acid can form ternary complexes with iron and aminoglycosides, leading to the formation of ROS (Lesniak et al., 2005). However, it is not clear which mechanism is the main source of ROS, and the formation mechanism of ROS remains to be solved.

**Figure 1:**
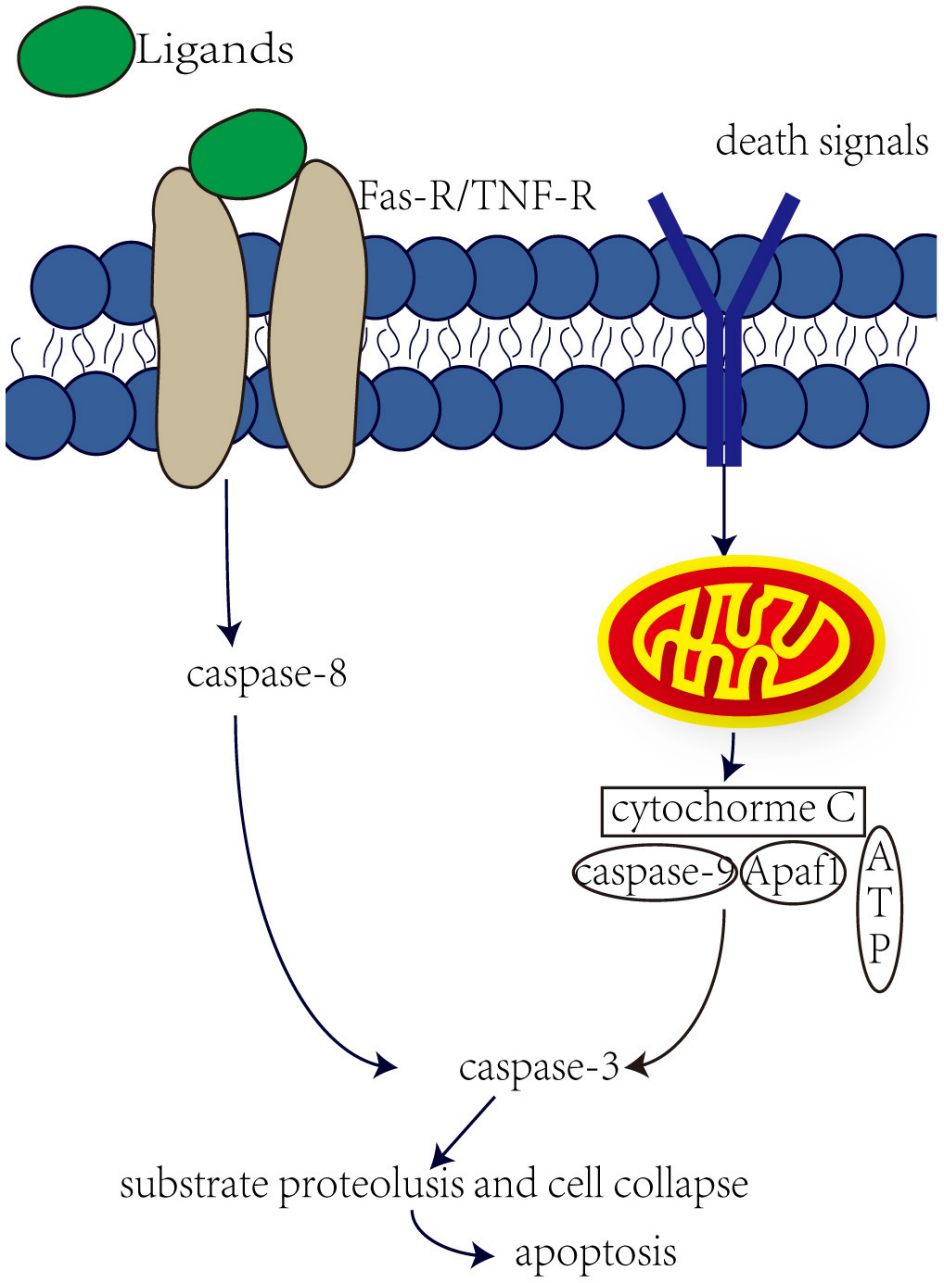
Internal and external pathway that induce apoptosis. The external pathway, the ligand binds to the death receptor to active it, which in turn induces a cascade of caspase-8 and caspase-3, leading to apoptosis. The internal pathway, some death signals, such as stress, DNA damage and faulty cell signaling, can induce mitochondria to release cytochrome c, which may form apoptotic bodies, which in turn activate caspase-3 and lead to apoptosis.

Aminoglycosides have both been reported to induce cell necrosis and apoptosis, but seem to be the main cause of cell apoptosis. Apoptosis is mainly regulated by the activation of caspase through internal or external pathways (Figure 1). In the internal pathway, mitochondria release apoptogenic factors into the cytoplasm to active caspase, in the external pathway, caspase is activated by ligand binding to death receptors (Rybak and Kelly, 2003). Studies have shown that aminoglycosides induce apoptosis through internal rather than external pathways. Perhaps in response to the production of ROS by aminoglycosides, the expression of anti-apoptotic Bcl-2 was decreased, and the expression of pro-apoptotic Bcl-X_L_ was increased, and Bcl-X_L_ was transferred into the mitochondria. This in turn leads to increased mitochondrial permeability, the release of apoptotic factors, and the possible formation of “apoptotic bodies” with Apaf1 and caspase-9. Downstream caspases are activated, such as caspase-3 and caspase-7, leading to substrate proteolysis and cell collapse. In aminoglycoside toxicity, overexpression of Bcl-2 prevents hair death and activation of caspase-9 (Cunningham et al., 2004). Meanwhile, after aminoglycoside administration, an increase in JNK components was observed, and JNK plays important role in mitochondria-mediated apoptosis. JNK can promote the release of cytochrome c. However, recent studies have shown that O2• from liver tumor cells and directly cause the release of cytochrome c through a voltage-dependent anion transport channels without damaging the mitochondrial membrane (Madesh and Hajnoczky, 2001). Therefore, ROS plays an important role in aminoglycosides-induced hearing loss.

## Prevention of Ototoxicity Induced by Aminoglycoside

In the course of clinical treatment, alternative drugs should be used as far as possible or to reduce ototoxicity. Patients treated with ototoxic drugs should be monitored for the prevention of ototoxicity due to medication. At present, medical personnel consider three ways to reduce the problem of drug-induced ototoxicity: development of efficacious ototoxic protective drugs; reversing ototoxicity-induced symptoms using neurotrophic grow factor; screening for genetic markers in patients at high risk of ototoxicity (for example, people who carry mutations in mtDNA such as A1555G or C1494T) (Ramma et al., 2019).

According to the mechanism of ototoxicity induced by aminoglycosides introduced previously, prevention of ototoxicity mainly includes two aspects: “upstream protection” and “downstream protection.” “Upstream prevention” refers to blocking of ROS formation or ROS scavenging, inhibition of nitric oxide synthesis, use of NMDA receptors antagonists, and increase of endogenous antioxidant enzymes. “Downstream prevention” refers to the inhibition of the downstream caspase cascade induced by ROS, as well as the inhibition of JNK cascade.

### Upstream Prevention

Studies have shown that inhibiting ROS formation and ROS scavenging can reduce aminoglycoside-induced ototoxicity. Because aminoglycosides can interact with iron to produce ROS (Lesniak et al., 2005), metal chelators may be used as protectors for ototoxic drugs. The study found that deferoxamine and the iron chelators 2.2′-dipyridyl had a protective effect against gentamicin-induced hearing loss (Dehne et al., 2002). This suggests that metal chelating agents can act as protective agents. Use of antioxidants can reduce ROS levels and effectively prevent aminoglycosides-induced ototoxicity. These include coenzyme Q10 (Fetoni et al., 2012), alpha-tocopherol (Fetoni et al., 2003), D-methionine (Campbell et al., 2016). ROS can be produced in a variety of ways, and mitochondria are the main source of ROS. What's more, ROS production can attack mitochondria. Intrinsic mitochondrial cell death pathway plays an important role in the process of hair cell death induced by aminoglycosides. Therefore, therapies that control mitochondrial homeostasis may be more effective in preventing aminoglycoside-induced hearing loss. Mitochondria-targeted antioxidants are superior in reducing mitochondrial oxidative damage (Dhanasekaran et al., 2004). Mitochondria-targeted antioxidants are expected to help prevent mitochondria-related diseases (Fujimoto and Yamasoba, 2019). Recent studies have shown that SS-31 peptides may be able to achieve mitochondria-targeted drug delivery to prevent aminoglycosides from damaging hair cells (Kuang et al., 2017). Therefore, the strategy of blocking the formation of ROS induced by aminoglycosides with metal chelators and eliminating ROS with mitochondrial targeted antioxidant drugs may effectively prevent the ototoxicity induced by aminoglycosides.

In addition, the nitric oxide (NO) synthesis inhibitor was found to have a protective effect against the ototoxicity of aminoglycoside. Dexamethasone acts mainly as a glucocorticoid, not a mineralocorticoid (Himeno et al., 2002). Dexamethasone can inhibit the increase of NO synthase mRNA, inhibit NO synthesis and free radical formation to protect OHC from the ototoxic effects of aminoglycosides (Himeno et al., 2002; Park et al., 2004).

Increasing evidence suggests that aminoglycosides may mimic the effects of polyamines on NMDA receptors (Puel, 1995). Aminoglycosides play a key role in ototoxicity by activating polyamine-like NMDARs. More importantly, overactivation of NMDARs produces NO, which induces oxidative stress on hair cells. Therefore, it has been suggested that NMDA receptors antagonists may prevent aminoglycoside-induced ototoxicity. Memantine is an NMDA receptors antagonist that reduces aminoglycoside induced hearing loss (Pavlidis et al., 2014). This suggests that NMDA receptors antagonists are a strategy for the induction of ototoxicity by aminoglycosides.

Under normal circumstances, ROS can be cleared by endogenous antioxidant enzymes (Kopke et al., 1999). Therefore, it has been proposed to increase the endogenous antioxidant enzyme pathway to prevent aminoglycoside-induced ototoxicity. A study showed that M40403, a superoxide dismutase mimetic, prevented gentamicin-induced ototoxicity (McFadden et al., 2003).

### Downstream Protection

**Figure 2:**
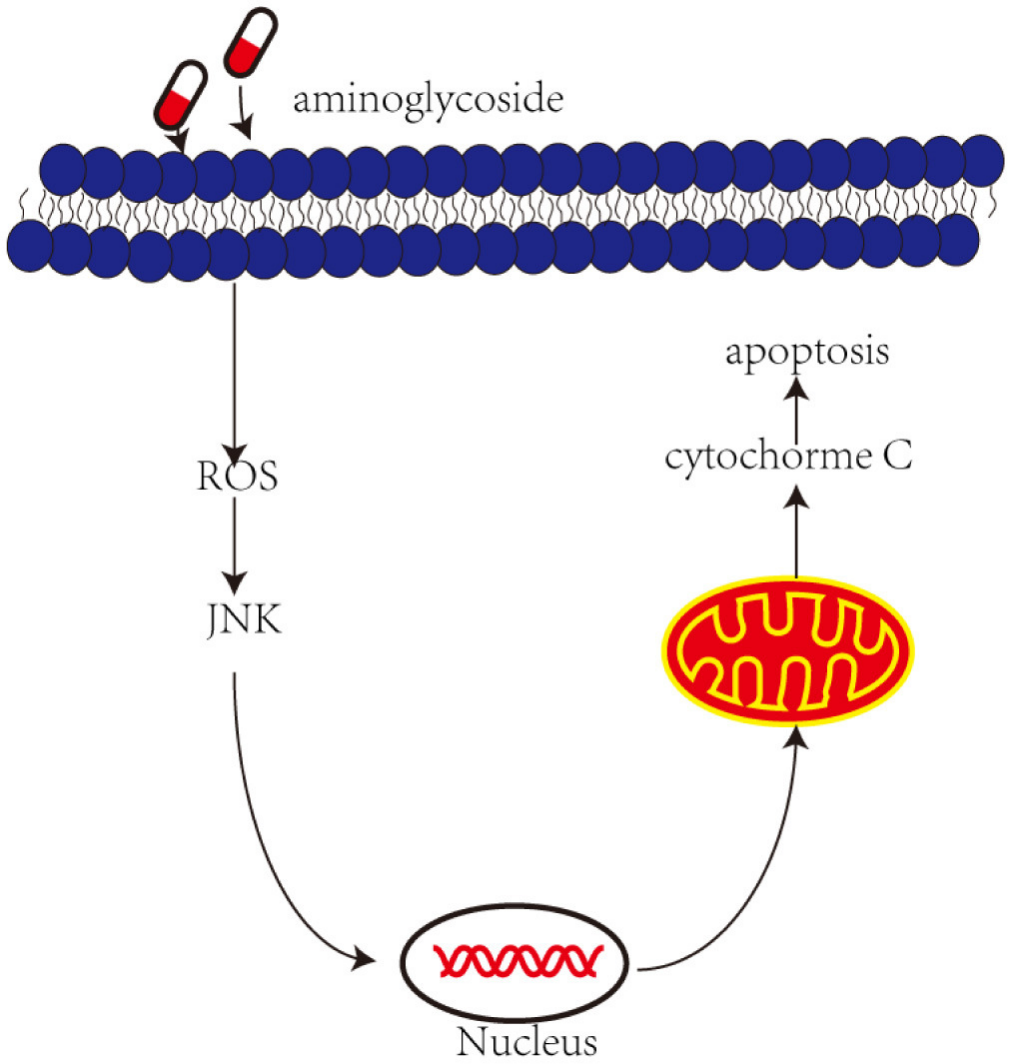
Aminoglycoside induce activation of JNK and then induces apoptosis. Aminoglycosides enter the outer hair cells, induce the production of ROS. In response to ROS and then activate JNK, they are translocated into the nucleus and activate some genes, which in turn induce mitochondria to release cytochrome c and induce cell apoptosis.

Recently, an increasing number of studies have shown that the administration of aminoglycosides leads to JNK activation and apoptosis of vestibular hair cells (Figure 2). The c-Jun N-terminal kinases (JNK) is a key member of the MAPK family and plays an important role in cell apoptosis. ROS may be upstream modulators of JNK activation, which may activate kinase cascades. Systemic administration of CEP-1347 (a JNK signal inhibitor) attenuates gentamicin-induced hearing loss and hair cell damage (Ylikoski et al., 2002). Another study showed that D-JNKI-1 (A synthetic inhibitor of JNK phosphorylation) protects against aminoglycoside-induced hair cell damage and hearing loss (Eshraghi et al., 2007). Therefore, blocking c-Jun N-terminal kinase can prevent the ototoxicity induced by aminoglycosides.

ROS induced by aminoglycosides can cause the release of cytochrome c in mitochondria and cascade activation of caspase, leading to substrate proteolysis and cell collapse. The release of cytochrome c is mediated by B-cell lymphoma-2 (Bcl-2) family. Studies have shown that overexpression of Bcl-2 in transgenic mice can reduce hair cell loss and prevent hearing loss after aminoglycosides administration (Cunningham et al., 2004). Caspase inhibitions (DEVE and ZVAD) attenuate the ototoxicity of gentamicin by blocking the activation of caspase-3 induced by gentamicin (Wei et al., 2005). What's more, minocycline attenuate ototoxicity better than the use of caspase inhibitors alone (Wei et al., 2005). In addition to inhibiting the activation of caspase-3, Minocycline may also inhibit phosphorylation of P38 MAPK and the release of cytochrome c (Wei et al., 2005). Therefore, it may be more effective to inhibit these ototoxic-inducing pathways together.

## Discussion

The above protective agents are based on animal studies and have a preventive effect on ototoxicity. However, there are some limitations in animal research and there are some problems in the transition from animal research to human clinical research. On the one hand, we need to find suitable protective agents for clinical use, so that the protective agents will not affect the antimicrobial effect of aminoglycosides. Studies have shown that D-methionine does not interfere with antimicrobial effects of tobramycin (Fox et al., 2016), but it is not clear whether other protective agents have any effects on the antimicrobial activity of aminoglycosides. On the other hand, because of the obvious differences in pharmacokinetics and drug elimination between animals and humans, it is not effective to calculate drug dosage by body weight. In addition to the above protective agents, aminoglycosides with low ototoxicity such as etimicin can also be used, which can effectively reduce ototoxicity (Yao et al., 2020). At the same time, new alternative drugs can be developed to reduce ototoxicity. Recent studies have purified hospital gentamicin, and analyzed the ototoxicity and antimicrobial activity of individual C-subtypes and impurities, providing ideas for the design of future drugs (O'Sullivan et al., 2020).

## Conclusion

Aminoglycosides, a class of clinically important drugs, are widely used worldwide against gram-negative bacterial infections. However, there is growing evidence that aminoglycosides can cause hearing loss or balance problems. In this article, we mainly introduce the main mechanism of ototoxicity induced by aminoglycosides. Genetic analysis showed that the susceptibility of aminoglycosides was attributable to mutations in mtDNA, especially A1555G and C1494T mutations in 12S rRNA. In addition, the overexpression of NMDA receptors and the formation of free radicals also play an important role. Understanding the mechanism of ototoxicity induced by aminoglycosides is helpful to develop new therapeutic methods to protect hearing. In this article, the prevention methods of ototoxicity induced by aminoglycosides were introduced from the upstream and downstream aspects. It has been shown that the use of some antioxidants and inhibitors of caspase can prevent cell apoptosis and effectively prevent aminoglycosides-induced hearing loss. In addition, the delivery of mitochondria-targeted drugs is of great significance for treatment. However, recent reports have found that autophagy may also play an important role in the induction of ototoxicity by aminoglycosides, and autophagy may be protective mechanism of hearing (He et al., 2017). Meanwhile, autophagy has been reported to play a role in hearing protection (He et al., 2020; Zhou et al., 2020; Liu et al., 2021). Autophagy may be another good way to prevent ototoxicity induced by aminoglycosides.

The mechanism of ototoxicity induced by aminoglycosides and prevention methods described above have been established from animal experiments and can be used as a potential means to prevent ototoxicity induced by aminoglycosides. We still have a lot of work to do in the transition from animal research to clinical use. The side effects brought by aminoglycosides to patients should not be ignored, and we should further develop new alternative or therapeutic drugs to treat hearing loss caused by ototoxic drugs.

## Author Contributions

XF, PW, and PL: writing-original draft, investigation, software, and writing-review and editing. JP, SG, YZ, YA, CY, and ZY: investigation. JG, JY, and JF: writing-review and editing, supervision, and administration. RC writing-review and editing, funding acquisition, and supervision. All authors contributed to the article and approved the submitted version.

## Conflict of Interest

The authors declare that the research was conducted in the absence of any commercial or financial relationships that could be construed as a potential conflict of interest.
